# An Epidemic of Drug Resistance: Tuberculosis in the Twenty-First Century

**DOI:** 10.3390/pathogens12050652

**Published:** 2023-04-27

**Authors:** Jens Seeberg

**Affiliations:** Department of Anthropology, Aarhus University, 8270 Hoejbjerg, Denmark; jseeberg@cas.au.dk

**Keywords:** tuberculosis, biosocial, global health, antimicrobial resistance

## Abstract

With an estimated two billion people being carriers of latent tuberculosis infection (LTBI), the gains achieved by increasing access to diagnostics and treatment, although substantial, have had a modest impact on the global burden of tuberculosis (TB). At the same time, increased access to treatment has had the unintended consequence that drug-resistant TB (DR-TB) has increased dramatically. Earlier TB control strategies strongly emphasizing medical treatment have failed to address these issues effectively. The current strategy to eliminate TB by 2050 is accompanied by a call for a paradigm shift, emphasizing patient rights and equity more. Based on ethnographic fieldwork in Odisha, India, and global-level TB conferences, this paper contrasts the dynamics of global health policy and strategy-making with the lived realities of patients with DR-TB. A more thorough rethinking of the biosocial dynamics that impact the pathogenic disease is required to develop a comprehensive paradigm shift for TB control in the twenty-first century.

## 1. Introduction

Broadly accepted estimates inform us that at least two billion people—25–30 percent of the world’s population—are carriers of latent tuberculosis infection (LTBI) [1]. According to WHO estimates, 10.6 million new cases of active tuberculosis (TB) infection developed in 2021 [2], which was a slight decrease from 11.2 million reported in 2000, as an estimated decline in incidence rate from 184 to 134 since the late 1990s was almost canceled out by the global increase in the human population. Furthermore, the syndemic synergies between the COVID-19 pandemic and TB led to an increase in estimated TB incidence in 2020–21 [2].

The modest progress in TB control should be assessed against substantial increases in funding for the global TB control program since 2000 and, in 2015, the introduction of a strategy to eliminate TB and the launch of a new ‘paradigm’ to achieve this end by 2030.

This paper discusses the proposed new paradigm on the basis of ethnographic studies and text analysis and compares the global health discourse as accessible through an annual world conference on TB and lung disease with the lived realities of people with drug-resistant TB (DR-TB) in India, using a case study as illustration. It argues that the shift in TB control presented as a paradigm shift does not sufficiently challenge the old approaches that have proven unsuccessful and the biosocial approach to TB control could contribute to achieving an actual paradigm shift.

### 1.1. Latent TB Infection

LTBI is unevenly distributed globally, which is more likely to be around forty percent in high-burden countries. Active TB disease manifests in five to ten percent of all LTBI cases and can, in theory, happen to any infected person but is usually facilitated by prior weakening of the immune system and follows the fault lines of exposure and late diagnosis that go together with poverty, high population density in poor neighborhoods and poor nutritional status.

Development of clinically manifest TB disease among LTBI carriers has some degree of predictability among so-called risk groups, such as those co-infected with HIV or living in close contact with TB patients, and treatment guidelines for preventive treatment released by the World Health Organization (WHO) in 2020 [3] recommended medical treatment for LTBI in such cases. However, the document also acknowledges that ‘other people at risk’ such as dialysis and silicosis patients, in addition to socially defined populations such as migrants from countries with a high TB burden, homeless people, prisoners, health workers, and people using drugs, may be screened and given preventive treatment (conditional recommendation) even if the effect is uncertain. In addition, children and adolescents with LTBI have a higher risk than adults for developing TB disease and have recently been recommended for preventive treatment [4]. Whereas the underlying paradigm thus recognizes the importance of living conditions among people with LTBI, the solution remains one of (preventive) medical treatment without addressing the conditions causing their increased risk of active infection.

### 1.2. Increase of Drug-Resistant TB Incidence

The overwhelming presence of LTBI is one of several reasons why the so-called DOTS (Directly Observed Treatment—Short-course) promoted by WHO since the mid-1990s has had limited success in addressing TB as a global health problem even if the strategy increased access to effective treatment for millions of patients. Another reason is its inability to effectively manage the growth of drug resistance that the strategy was supposed to prevent. Whereas the overall trendline for the global incidence of TB has been slowly declining, and a similar trend for DR-TB in Europe has affected the global trend positively [2], in most high-prevalence countries, DR-TB has increased. However, despite improvements in the global surveillance of DR-TB [5], the quality of statistics is subject to several biases. It depends on case detection, with large groups of patients never being diagnosed. To add to this problem, there is a growing concern that drug resistance is also increasing among the world’s LTBI cases [3], threatening to undermine current LTBI regimens in the future. As DR-TB is a consequence of medical treatment, a brief introduction to the recent history of global health efforts to control TB through treatment-based interventions is provided below.

### 1.3. Directly Observed Treatment—Short-Course (DOTS)

Until the 1990s, TB was, in many countries, a neglected disease. In the rich countries, it had disappeared as a public health priority, hidden from political attention in marginalized populations such as the homeless, migrant workers, and poor neighborhoods with little access to healthcare services. In low-income countries, funding for healthcare and other essential deliverables of the state had been severely reduced due to so-called restructuring programs imposed by the World Bank and International Monetary Fund (IMF), often in collaboration with dysfunctional forms of governance characteristic of postcolonial power structures. In this process, TB came to be seen as a tropical disease. Unlike other conditions in this category, such as malaria and other vector-borne diseases that were considered ‘tropical’ for ecological reasons, TB was pushed into this category due to its relative disappearance from the global north. However, due to its syndemic dynamics with human immunodeficiency virus (HIV), it reappeared on the radar of rich countries, and in 1993, WHO declared TB a global public health emergency [6]. This call for attention followed the introduction in 1991 of the so-called DOTS strategy, which was in need of substantial funding for global implementation.

DOTS is an acronym for Directly Observed Treatment, Short-course, ‘short’ referring to six months. It was a highly complex health intervention [7] that required simultaneous interventions at many health system levels and depended on the quality of the existing health infrastructures despite its vertical design. ‘Directly observed’ meant that the patient was in contact with a so-called DOT-provider (DP), and they then had to visit the DP and take medicine in front of this person. The DOTS approach was developed in response to problems with patient adherence to earlier unsupervised treatment regimens of even longer duration. As has been pointed out by Ogden and others, DOTS could be criticized for moving away from ‘models of communication and co-operation between providers and patients’, ‘back to a traditional medical approach with the patient as the passive recipient of advice and treatment’ [8], representing a paternalistic and context-free response to the challenge of premature interruption of treatment [9].

In addition to directly observed treatment (DOT), the concurrent need for access to diagnosis through sputum microscopy, uninterrupted availability of high-quality drugs for the entire treatment period of each patient, and a comprehensive monitoring system resulted in a highly complex health intervention that required considerable political backing at all levels. In a high-burden country such as India, which accounted for around 40% of the world’s notified TB cases in 1991 [10], the backdrop against which DOTS was implemented was a poorly functioning TB control program that, according to an evaluation, ‘suffered from managerial weaknesses, inadequate funding, an over-reliance on x-ray for diagnosis, had frequently interrupted supplies of drugs, and low rates of treatment completion’ [11] (19). There was an urgent need for policy change, and WHO advocated DOTS as an answer to the problem.

By this time, the increased access to treatment was celebrated as a success. DOTS was based on a calculation that with a case detection rate of 70% of all actual cases and a cure rate of 85%, the TB caseload could be cut by half within a decade [12], using combination therapy with four different types of antibiotics, which were given during an ‘intensive phase’ of two months followed by a ‘continuation phase’ of additional four months. Also, being a multi-drug regimen, it was intended to control the risk of developing drug resistance. Whereas DOTS represented an improvement in TB treatment relative to the earlier neglect situation, it soon became evident that increased access came with the cost of growing drug resistance. In contrast, the hoped-for decline in the incidence rate was barely visible. Patients with relapse after treatment or where treatment had been interrupted for at least two months were routinely placed on a different regimen including injectable Streptomycin, but as pointed out elsewhere [13], access to drug susceptibility testing during the 1990s and 2000s had been minimal.

India is a high-prevalence country with a significant disease burden attributable to TB, accounting for around 25% of TB cases worldwide. In India, DOTS was launched in 1993 and was gradually scaled up from an initial coverage of 2.35 million people to nationwide coverage by 2006. Towards the end of the 2000s, the upgrading of laboratories and availability of medicines for patients who tested positive for drug resistance began to slowly change the Indian treatment scenario. This also led to dramatic changes in the figures involved: an estimate of 99,000 new multidrug-resistant TB (MDR-TB) cases annually by 2011 was quoted by a leading national expert [14], whereas only 4217 patients had been placed on relevant treatment by the end of 2009, of whom only 756 were alive 12 months later.

### 1.4. Revising the TB Control Strategies

The problem of the rising number of DR-TB cases and the need for urgent action had been pointed out as early as the late 1990s. Farmer and colleagues [15,16] argued in favor of individualized treatment regimens for MDR-TB. Realizing the alarming situation but insisting on standardized treatment for resource-poor settings, WHO developed guidelines for the so-called DOTS+ strategy [17] as a first step towards programmatic treatment of DR-TB, launched as the ‘STOP TB‘ strategy that would cover the period 2006–2015. In addition to standardized treatment for DR-TB, the strategy would strengthen collaboration with HIV services and develop new diagnostics “supported by a budgeted plan with feasible targets” [18]. DOTS was still considered a success in terms of increased access to TB treatment in lower-income countries, even if the somewhat optimistic prediction regarding its ability to reduce TB prevalence and prevent drug resistance was proven wrong. Nevertheless, STOP TB was launched with similar optimism. With the Stop-TB strategy, “by 2015, global TB incidence could be reversed and its prevalence and mortality reduced by half compared to 1990” [18].

Realizing the limitations of DOTS and the growing problem of DR-TB, WHO and other global health actors engaged in TB control have regularly modified the TB control strategies during the past twenty years, eventually launching the ‘End TB Strategy’ in 2014 [19]. At the same time, the UN-hosted Stop-TB partnership called for a paradigm shift away from a near-exclusive focus on medical treatment and towards so-called patient-centered and community-oriented approaches to TB control. A central document describes this paradigm shift in predominantly ‘social’ (as different from ‘medical’) terms, e.g., ‘Medical interventions alone will not be enough to end TB. Nonmedical actions and investments, such as improved housing and sanitation, poverty reduction, and strengthened social safety nets will drive down the number of people becoming ill and dying from TB’ [20]. At the same time, the new strategy calls for integrated health systems, including integrating TB interventions with HIV/AIDS and maternal and child health programs. These reorientations can perhaps be seen as an attempt to consider the syndemic potentials and well-established synergies with other epidemics and socioeconomic and other inequalities in global TB control, even if the concept of syndemic is absent from the strategic documents. Based on ethnographic fieldwork, this article discusses this intended, if slow, reorientation away from a vertical program characterized by a dominant biomedical approach towards a broader acknowledgement of the implications of understanding TB as predominantly a ‘social disease’. While acknowledging that global TB control is currently in the process of change, I shall argue that the intentions of the paradigm shift are not far-reaching enough and that understanding the biosocial and syndemic dynamics driving the increasingly drug-resistant tuberculosis pandemic may require a re-thinking of the scientific basis of the paradigm underlying TB control.

## 2. Materials and Methods

Materials presented here are constituted through multi-sited ethnography, with one site being the annual Union Conference during 2014–2018 and another being two districts in the Indian state of Odisha. The purpose of presenting findings across these different contexts is to juxtapose global health policy discourse with the lived experiences of DR-TB patients in light of the proposed paradigm shift that emphasizes patient-centered treatment.

Conference ethnography is a new approach in anthropology but recent contributions point to conference spaces as epistemic cultures [21] in addition to being essential venues for networking [22]. The conference material consists of notes and recordings from selected presentations, observation of meetings and informal interaction, interviews with key informants, including industry representatives, collection of conference merchandise, photographs, and abstract books available on the Union’s website. The author presented as part of a panel on WHO ethical guidelines. In the present paper, observations conducted during conference participation and text analysis of all conference abstracts from the period 2014–2022 (*N* = 1281) have been included.

The main body of the Odisha material consists of 111 h-long interviews with people living with DR-TB. Interviews were supplemented with observations, photos, 83 diaries of five participants who were able to engage in this activity, and interviews with medical doctors and TB officers at various levels. The interviews were conducted during repeated home visits during 2015–2019 and additional online interviews were carried out in 2020 during the COVID-19 pandemic. All interviews were audio-recorded, transcribed, and all qualitative data were analyzed using Nvivo software version 1.0 (QSR International, Burlington, MA, USA). The project was carried out in collaboration with a local NGO working for the rights of TB patients. Whereas the limits of this article do not allow for a presentation of the study in its entirety within, a case study illustrates challenges typically faced by people living with DR-TB in resource-poor settings.

## 3. Results

### 3.1. Annual Conference of Union against Tuberculosis and Lung Disease

The future, the present, and the past of the synergetic dynamics of tuberculosis and its many syndemic interactions come together at the annual World Conference of the Union Against Tuberculosis and Lung Disease (hereafter, the Conference and the Union, respectively). The Conference is a leading forum for the discussion of the latest research, policies, and practices related to tuberculosis and lung health. Over the years, the Conference has covered a wide range of topics, from basic research on the biology of tuberculosis to clinical trials of new drugs and vaccines, and from public health policies to the social and cultural factors that influence the transmission and treatment of the disease.

Here, global health actors such as WHO and the Bill and Melinda Gates Foundation, pharmaceutical and biotech companies involved in TB treatment and diagnostics, program managers, policymakers, representatives of nongovernmental organizations, and researchers meet to be updated and inform each other on the latest treatment regimens, diagnostic technologies, vaccine development, and epidemiological statistics, together with discussions of patient rights, drug resistance, and operational research findings from interventions seeking to improve TB care. The exhibition area was each year a central area of the conference, visually dominated by the biomedical industry. A special arena in the margin of the main exhibition area would be allotted to ‘community representatives’, where TB activists and patient organizations, who had been able to find the means to cover the conference fee, would have a space.

The 2014–2018 period was characterized by several important events that influenced global TB control and the Conference provided a window where these developments could be followed. Three events were of particular significance: (1) The launch by WHO of the Strategy to End TB (achieve elimination) at the conference in Barcelona in 2014, and the subsequent call by the STOP TB Partnership for a ‘paradigm shift’ in global TB control at the 2015 conference in Cape Town. (2) New medicines for DR-TB, such as Bedaquiline and Linezolid were identified resulting in reduced mortality rates and a shorter treatment period for DR-TB. Lastly, (3) molecular diagnostic tools for DR-TB were introduced, notably the GeneXpert system produced by Cepheid, located in Sunnyvale, CA, USA, could test for Rifampicin-resistance within hours instead of weeks, hence potentially increasing access to DR-TB diagnostics substantially. Whereas improved diagnostics and shortened treatment regimens were also key to the TB elimination strategy, the paradigm shift entailed a move from the paternalism of DOTS to a human right-based and patient-driven approach that would take the social drivers of the TB pandemic more seriously.

**Figure 1:**
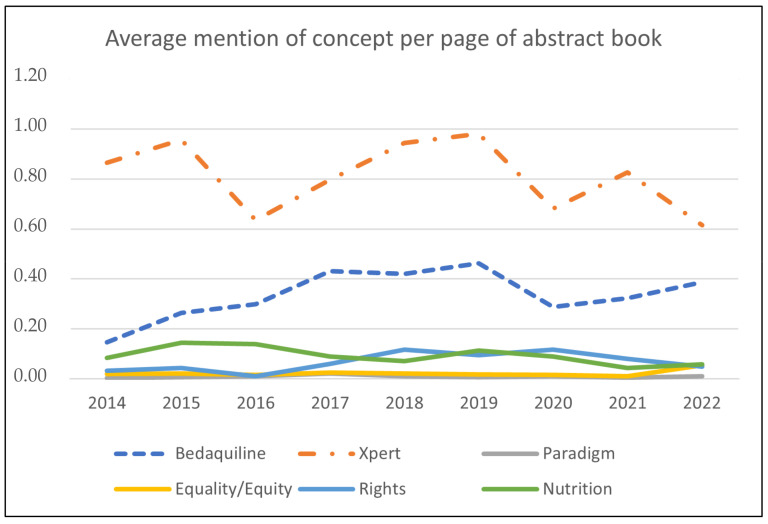
Presence of selected keywords per page in Abstract Book of the annual Union World Conference, 2014–2022. Source: https://theunion.org/our-work/conferences/history-of-the-union-world-conference-on-lung-health/conference-abstract-books (accessed 19 March 2023).

The many presentations at the conference provide a window into investments in research, education, and implementation activities, which were published in the abstract book of the conference. Therefore, a paradigm shift would likely be reflected in the dominant discourse constituted by the myriad of presentations at the Union World Conference during the period where this paradigm shift was to take place. Figure 1 presents the results of a text search in the published abstract books, shown as an average number of hits per page by year for the following keywords: (1). Xpert; (2). Bedaquiline; (3). Rights; (4). Equality or Equity; (5). Nutrition; and (6). Paradigm. The number of pages varied between 451 in 2021 and 640 in 2019, totaling 4789 pages. Whereas the page was the unit of analysis, each page contained 2–5 abstracts, with an estimated total exceeding 15,000 abstracts. Keywords 1 and 2 represent a standard biomedical focus, whereas keywords 3–5 are used as indicators for introducing the new patient-centered paradigm. Keyword 6 would express discussions of the paradigm shift itself. As a hypothesis, it could be expected that keywords 3–6 would become more visible in the discourse after 2015, whereas the presence of keywords 1 and 2 would be relatively independent of the new strategy even if integrated into it due to their clinical relevance.

This is a crude measure of the degree of success with which the intended paradigm shift has effectively reoriented the activities reported at the conferences, and it does not take into account that different abstracts and presentations have different weights outside the conference space. Nevertheless, it is noteworthy that policy translation of the new strategy with its call for a new paradigm needs to be more visible in the global reporting of research, policy development, and applied interventions at these conferences.

### 3.2. Living with DR-TB

#### 3.2.1. General Findings

104 in-depth interviews were carried out during repeated home visits with 20 adults with DR-TB in two districts in Odisha, India, 2015–2019, with follow-up online interviews in 2020 during the COVID-19 pandemic. Five participants wrote a diary for parts of the period, depending on their well-being and capacity. Table 1 provides an overview of sex and age distribution among the participant.

Out of 20, nine had completed treatment within a five-year period, seven had died, two had moved elsewhere and could not be followed, one participant who also had diabetes was still under treatment after five years, and one female participant stopped treatment due to adverse effects and pursued Ayurvedic treatment instead (Table 2). She was symptom-free at the end of the study (for a detailed case description, see [23]).

**Figure 2:**
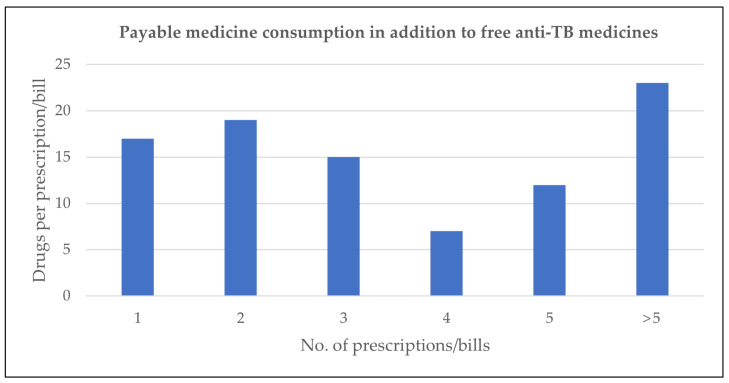
Number of payable drugs prescribed per prescription/bill audited.

Twelve participants had maintained medical records, including prescriptions and medical bills. A total of 93 prescriptions/bills were analyzed. Figure 2 shows the number of drugs per prescription/bill.

The majority of medicines were ancillary drugs, as anti-TB drugs were provided by the hospital in most cases. On average, participants purchased four medicines per prescription/bill, representing substantial and long-term out-of-pocket expenditures. In addition, the records documented that participants routinely had to pay for diagnostic tests other than sputum tests e.g., blood tests, x-ray, and urine examinations.

Whereas delivery of medicines for DR-TB was organized through the public healthcare sector, private healthcare delivery plays a major role in TB treatment in India, as medical doctors employed in public hospitals often run a private clinic outside the hospital, and they prescribe tests and medicines to be procured from the market.

**Figure 3:**
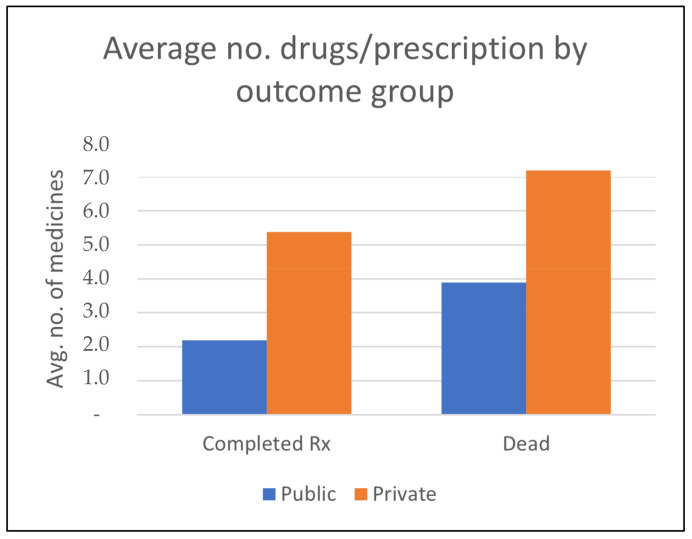
No. of drugs per prescription by public vs. private providers for two main outcome groups.

For the two main outcome groups, consumption of medicine was substantially lower for those who completed treatment than for those who died during treatment (Figure 3) for both private (out-of-pocket) and public (for free) treatment.

#### 3.2.2. Case Presentation

Batuk was 19 years old and in the tenth grade when he was diagnosed with TB. His DOT-provider was a relative who would deliver the medication and leave without checking that he was taking it. He did at first but stopped when he felt better and concentrated on his marriage plans. After 3–4 months without medication, he started coughing up blood. When he returned to the hospital, he was severely reprimanded. He was labeled a ‘defaulter’—a person who does not fully adhere to the treatment regimen and is therefore attributed with the same negative qualities as a bad payer. He was simultaneously diagnosed with DR-TB and the doctor marked his file with the words: ‘chronic defaulter’.

While some patients found it challenging to adhere to the treatment regimen for drug-susceptible TB of four standard antibiotics, adhering to the DR-TB regimen was even more so. It involved ten to sixteen daily tablets and injections during the intensive phase of six to nine months, and fewer drugs during the 18-month continuation phase. The treatment was associated with a high risk of liver and kidney damage, the risk of permanent hearing loss, and other side effects. Perhaps comparable to oncological chemotherapy, DR-TB treatment differed in that the drugs were administered daily, for two years, and by the patient, who, except for injections to be administered at the health center, had to manage the treatment on their own, often dividing the day between sleeping, taking the drugs and vomiting.

In Batuk’s case, DR-TB was probably caused by irregular treatment at the age of 19, at a time when he was preoccupied with marriage and family conflict. When we first met him at the age of 22, he was struggling with the side effects of his medication and the illness of his young daughter. He lived with his wife and daughter in a one-room house next to his brother’s and parents’ tiny houses in a slum in a big city, squeezed between a rubbish dump, an open sewer, and a cowshed. Batuk’s diary provided a glimpse of a life punctuated by the monotony of waking up, eating some of the little food available, taking medicine, vomiting, feeling sick, sleeping, waking up, going to the health post for injections, returning sick from adverse effects, arguing with family members about his inability to work, and the constant need for money. This narrative was interspersed with moments of playing with his daughter and worrying about her. However, occasionally, no matter how sick he was, Batuk would take another sick family member to the hospital or pick up medicine for them at the pharmacy. His illness had made him a local expert on interacting with the health system as well as a critical expert because he was acutely aware of the catastrophic cost of his illness to the household and equally critical of the extraction of money from poor patients that he witnessed for himself and others—even though all drugs and tests for DR-TB patients were supposed to be free. In his words:

“Going to the big hospital, (the doctor) writes for injection for (INR) 500, 1000 (EUR 6.5; 13) one, one injection…… for one needle. This injection, for 8–10 days that you have to stay. Before it was free, now they say ‘for how many days you stay here, buy the syringe’…… ‘buy the needle’ (…) In 2013 and 2014 everything was free, but now in 2015 they tell [me] to buy everything…… if we don’t buy then they make us leave…… for all the reports it’s [INR] 500, 1000, 1500, 2000, 5000. So they say”.

Often, patients were sent to private clinics near the hospital for additional tests that were unavailable at the government hospital. In such cases, poor patients such as Batuk were entitled to reimbursement of the incurred expenses. However, patients often struggled in vain to get reimbursement. Batuk only received it in part several years into his treatment period and after complaining about his desperate situation on social media.

Batuk related that he often faced irregular drug supply during treatment for DR-TB, either because of delivery problems or staff absenteeism at the delivery point. During hospital admission, he had tried to get an explanation as to why medical students who filled in for more experienced doctors sometimes changed his regimen. On one occasion, when collecting medicines for the coming week, he had taken medicine supplies for several weeks himself to protect against treatment interruption, as nobody was there to hand over the medicines. However, his condition deteriorated, and in January 2016, he was hospitalized again, this time diagnosed with extensively drug-resistant TB (XDR-TB). While DR-TB patients were entitled to free hospital treatment, the reality was that they had to pay for most of their needs other than anti-TB drugs. This was followed by a battle, supported by a local NGO, to gain access to the new drug Bedaquiline, which had initially been approved for use only in certain states in India, excluding the state of Odisha. Finally, in January 2017, following a case brought by a TB patient in the Delhi High Court, the way was cleared for wider use [24]. However, it would take another 15 months and a direct appeal to the Chief Minister of Odisha on Twitter before Batuk became the first patient in the state to access Bedaquiline in March 2018. Despite the hope this brought, he died of TB in October 2020 after six and a half years of treatment. After his death, his wife Rubina and child were expelled from home and moved to Rubina’s parents’ house.

## 4. Discussion

The discussions at the Union World Conference and the treatment trajectory of patients such as Batuk, very different as they seem, concern the same issues: access to diagnostics and treatment, patient rights, equity, and sufficient nutrition. In very different ways, however, they point to the challenges of reorienting from a medico-centric paradigm to one where a thorough understanding of the social drivers of disease progression, development of drug resistance, and the concurrent need for and limitations of new biomedical technologies, is translated into successful TB management.

At the global policy level, the analysis of the conference presentations showed that the new paradigm was not visible in the contents of discussions as measured through the use of central keywords to define what was new in the new paradigm, even if abstracts were included up to the year 2022. In contrast, the focus on the development of technological innovation remained high. One visible reason for this is the role of industry in global health. The money flows in global health prioritize the products that can be sold and distributed through global health infrastructures, and the marketplace at the conference is a glimpse into a field in which social actors fight over resources in the form of different kinds of capital [25,26]. The forms of capital involved make the field uneven. Companies such as Cepheid (producer of GeneXpert) and Janssen (producer of Bedaquiline under the brand name Sirturo) have grown and gained from entering the field of DRTB. In contrast, other social actors such as the Indian pharmaceutical company Lupin have consolidated its position as a producer of generic drugs used for drug-susceptible TB. Other companies use the conference to attract attention to their products from global funders such as Gates Foundation, hoping they will become the next success story. Bill and Melinda Gates Foundation invested heavily in Cepheid to reduce the cost of cartridges required to perform the diagnostic test (Source: https://www.gatesfoundation.org/ideas/media-center/press-releases/2012/08/publicprivate-partnership-announces-immediate-40-percent-cost-reduction-for-rapid-tb-test accessed on 24 March 2023), thereby also boosting the value of the company’s stocks. Public investment in the development of Bedaquiline has been estimated to exceed that of Janssen by a factor of 1.6–5.1 [27], indicating a development where “organizing principles of state-centric pastoral and clinical health care have given way to speculative, market-driven approaches to health.” [28] (S307). So, to return to the World Conference marketplace, some social actors in the playing field have billions of dollars invested in the decisions and discussions that surface during such meetings. Other actors in this field depend on more modest donations and volunteers to promote the focus on human rights and equity. Whereas this focus is solely directed at health promotion, it presents a poor business case as it depends on investments in and promotion of transparent and rights-based governance and access to common goods such as universal healthcare and education in the nation-states of the global south.

The ethnographic material describing the challenges DR-TB patients face in Odisha illustrates why this is so. The case study highlighted the struggle for patient rights that Batuk felt were denied him unless he mobilized support through social media. Other patients could not do so when faced with out-of-stock medical supplies, high out-of-pocket expenditures for treatment and diagnostics that were supposed to be free, and insufficient support when facing severe adverse effects. On several occasions, the project team was ethically obliged to interfere by appealing to health providers in charge of TB care to ensure rightful treatment when patients could not manage their situation. Patients also struggled to navigate the sick role as, in most cases, they were breadwinners, and their prolonged disease created a financial crisis at the household level and pressure on them to work even if ill. Many households faced catastrophic illness expenditures because of the high involvement of the private sector in disease management during years of reduced income. During such crises, treatment expenses competed with daily expenditures for basic food. This caused interruptions of treatment and windows of opportunity for the pathogen to develop resistance to additional drugs.

Improvements in treatment and diagnostics, such as Bedaquiline and GeneXpert (or the Indian-produced equivalent device known as CBNAAT), are needed but insufficient. They provide important short-term solutions to improving case detection and drug-resistance. They offer shorter treatment duration and increased survival for multi-drug resistant TB patients until the pathogen has developed resistance. However, Bedaquiline resistance has already been reported in several studies [29,30], as has cross-resistance to Clofazimine, a drug repurposed for treating DR-TB [27,31].

The call for a paradigm shift in global TB control is not new. Social scientists had pointed to this need even before the DOTS strategy was launched and throughout the various subsequent strategies [8,32,33,34,35,36,37,38,39]. Whereas these contributions have called for the inclusion of social science knowledge and life science know-how, they have yet to challenge the separation of these knowledge domains in the first place. Others see the paradigm shift mainly in the form of rolling out therapeutic and technological innovations more effectively while lamenting that most high-burden countries fail to do so [40] without much reflection on why this is the case.

Hoping for a paradigm shift by adding social components to a failed paradigm appears insufficient. The repeated optimism of each new strategy is unfounded in the ground realities of the unfolding pandemic. This is apparent against the global LTBI prevalence described in the introduction. With 25% of the human population being carriers (in addition to unknown reservoirs in animals, including human-to-cattle DR-TB infection [41]), TB is a pathogen with enormous synergistic potential in relation to other diseases. Some of these also constitute epidemics such as HIV and diabetes, resulting in, e.g., TB-HIV and TB-diabetes syndemics [42,43,44,45,46,47]. In contrast, others involve devastating co-morbidity between TB and, e.g., cancer [48] or rheumatoid arthritis [49]. These inter-pathogenic dynamics are positively influenced by particular social configurations in which human hosts are embedded. The same is true for the biochemical communication that unfolds between medicinal substances, the human organism, its microbiome balances, and the pathogen targeted with medicines, such as *Mycobacterium tuberculosis*.

Such dynamics are partially recognized in the current focus on identified vulnerable populations or risk groups, such as people living with HIV/AIDS, close contacts of TB patients, or prisoners. However, the list is much longer and includes refugees, people living in conflict and war zones, people working in unhealthy environments in a large number of industries, smokers, people subject to heightened air pollution, and under-nourished populations, to mention some. As much as each person who develops active TB disease should have the right to timely diagnostics and medical treatment, they should also have the right to healthy living conditions that could prevent TB from progressing to manifest infection. A strategy that pursues TB control through the medicalization of poverty and impoverished work and living conditions is unlikely to succeed.

A paradigm shift in the context of scientific knowledge according to Kuhn [50] follows a period of crisis in which old assumptions and methods are challenged and need to be replaced by new ones. The brief history of TB control since the 1990s provided in the introduction points to such a crisis. However, according to Kuhn, a paradigm shift involves a fundamental shift in scientific thought and practice, in which the old paradigm is replaced by a new one that fundamentally changes the way that problems are approached and solved. In contrast, what is labeled as a paradigm shift by WHO builds on existing TB control strategies with medical treatment at the center while new priorities and methods are added. The WHO’s paradigm shift does not recognize the crisis of TB control as involving the basic understanding of TB. However, if TB infection is seen as primarily a biosocial response to dynamics of poverty, inequity, and other adverse life conditions, a new paradigm for TB control for the twenty-first century cannot merely be based on a revived social medicine. The global TB control program needs to develop an actual paradigm shift based on the scientific de-separation of the social and the biological [51,52] that will enable an understanding of the entanglements of the lives of microbes including pathogens such as TB and the lives and life circumstances [53] of their hosts in order to reconfigure relevant prevention approaches in a world where LTBI is part of the human condition.

## Figures and Tables

**Table 1:** Age and sex distribution among participants with DR-TB.

Age Group	Female	Male	Total
18–19		1	1
20–29	4	6	10
30–39		3	3
40–49	3	2	5
60–69		1	1
Total	7	13	20

**Table 2:** Treatment outcome as of the end of 2020.

Treatment Outcome	Female	Male	Total
Dead	3	4	7
Stopped treatment	1		1
Continued treatment after 5 years		1	1
Unknown		2	2
Completed treatment	3	6	9
Total	7	13	20

## Data Availability

Qualitative data cannot be publicly available for ethical reasons, as adequate anonymization cannot be achieved. Other materials used are publicly available.
